# Chidamide Maintenance Therapy Following Induction Therapy in Patients With Peripheral T-Cell Lymphoma Who Are Ineligible for Autologous Stem Cell Transplantation: Case Series From China

**DOI:** 10.3389/fonc.2022.875469

**Published:** 2022-06-07

**Authors:** Wei Guo, Xingtong Wang, Jia Li, Xianying Yin, Yangzhi Zhao, Yang Tang, Anna Wang, Ou Bai

**Affiliations:** Department of Hematology, The First Hospital of Jilin University, Changchun, China

**Keywords:** chidamide, PTCL, maintenance therapy, ASCT ineligible, induction therapy

## Abstract

**Objective:**

To assess the potential benefit of chidamide maintenance therapy after induction treatment in peripheral T-cell lymphoma (PTCL).

**Materials and Methods:**

The clinical data of 48 transplantation-ineligible patients with different PTCL subtypes who received continuous chidamide treatment after first-line therapy were collected. Progression-free survival (PFS), overall survival (OS), and safety were analyzed.

**Results:**

In total, 68.8% of patients were male (33/48), the median age was 59.5 years (22~80). The pathological subtypes were angioimmunoblastic T-cell lymphoma (AITL, 43.8%), anaplastic large cell lymphoma (ALCL, 16.6%), PTCL-not otherwise specified (NOS, 25%), NK/T-cell lymphoma (NKT, 14.6%). 35.4% (7/48) patients had intermediate or high risk (IPI=3~5). 20 patients (41.7%) received chidamide as a maintenance treatment after achieving a complete response (CR). 57.1% (16/28) exhibited a better response after chidamide (9 cases partial response [PR] to CR, 7 from stable disease [SD] to PR). The CR and overall response rate (ORR) were 60.4% and 93.8%, respectively. In addition, 21/21 AITL, 10/12 PTCL-NOS, and 8/8 ALCL, 6/7 NK/T exhibited CR/PR as the best response during the follow-up period. Meanwhile, the CR and ORR did not differ by age (<60 *vs* ≥60: 50.0% *vs* 70.8%, P = 0.091; and 91.7% *vs* 95.8%, P = 0.551). The median follow-up period was 12.8 months (3.0–66.6), 14 patients developed PD (29.2%), 10 of them died of lymphoma (20.8%). Totally, the 40 cases achieved CR/PR from 1st line regimen got better PFS as well as OS than the rest 8 cases (the 1-year PFS was 80.8% *vs* 46.9% and the 2-year PFS was 71.9% *vs* 46.9%, P=0.012. the 1-year OS was 89.9% *vs* 72.6% and the 2-year OS was 85.9% *vs* 48.6%, P=0.032). No patients discontinued treatment because of adverse events. The most common toxicities were neutropenia (75.0%), anemia (79.2%), thrombocytopenia (58.3%), and anorexia (45.8%), and fatigue (43.8%).

**Conclusion:**

Chidamide maintenance therapy led to improvements of PFS and OS with a manageable safety profile in patients with PTCL. Further randomized studies are required to examine the role of chidamide maintenance therapy in PTCL.

## 1 Introduction

Peripheral T-cell lymphomas (PTCLs) describe a rare and heterogeneous group of mature T-cell and natural killer cell lymphoid malignancies. They account for 10%–15% of all non-Hodgkin lymphomas worldwide, and the incidence in China is relatively high (approximately 25%) (1–3). Most patients receive cyclophosphamide, doxorubicin, vincristine, and prednisone (CHOP) or CHOP-like regimens as the initial therapy. Although most patients respond to treatment (4), these regimens are associated with high rates of relapse. It was reported that with the exception of ALK-positive anaplastic large cell lymphoma (ALCL), PTCLs are associated with a poor prognosis, with an estimated 5-year overall survival (OS) rate of 30% (5), Moreover, patients with relapsed or refractory PTCL have an extremely poor median OS of 5.8 months (6). Therefore, there is an unmet medical need for continuous therapy to maintain remission in PTCL.

In recent years, two histone deacetylase (HDAC) inhibitors, romidepsin and belinostat, were approved by the US Food and Drug Administration for the treatment of refractory or relapsed PTCL. In addition to its application in salvage regimens, one clinical trial found that continuing treatment with romidepsin in patients with stable disease (SD) could provide clinical benefits, supporting the application of HDAC inhibitors as maintenance therapy in PTCL. In the clinical trial which was conducted in relapse/refractory patients as mentioned above, romidepsin was used as salvage therapy and continued in those with SD or better response (7).

Chidamide is a novel member of the oral benzamide class of HDAC inhibitors exhibiting *in vitro* activities against a wide array of neoplasms. Based on the results of the pivotal phase II trial of patients with refractory or relapsed PTCL (N = 79), chidamide was approved in 2014 by the Chinese Food and Drug Administration for the treatment of refractory or relapsed PTCL (8–10). As an epigenetic modulator, chidamide induces growth arrest and apoptosis in tumor cells and enhances cellular antitumor immunity (11, 12). This provides a solid rationale for chidamide maintenance therapy.

The aim of this study was to assess the potential benefit of chidamide maintenance therapy after induction or salvage treatment in patients with PTCL.

## 2 Materials and Methods

Chidamide was added as consolidation therapy after PR1 or SD1 after evaluation.

### 2.1 Study Design and Participants

This was a retrospective, single center, single-arm study. Patients were eligible if they were 18–80 old with different PTCL subtypes according to the WHO 2016 classification including angioimmunoblastic T-cell lymphoma (AITL), ALCL(ALK+ or ALK-), PTCL-not otherwise specified (NOS) and NK/T cell lymphoma. Other eligibility criteria were previous induction treatment (P-GemOx for NK/T, CHOP or CHOPE for other subtypes), ineligibility for high-dose chemotherapy and autologous stem-cell transplantation, Eastern Cooperative Oncology Group performance status of 0–2, and Ann Arbor stage II–IV at diagnosis. All patients were required to have achieved a complete response (CR) or partial response (PR) after induction treatment.

**Figure 1:**
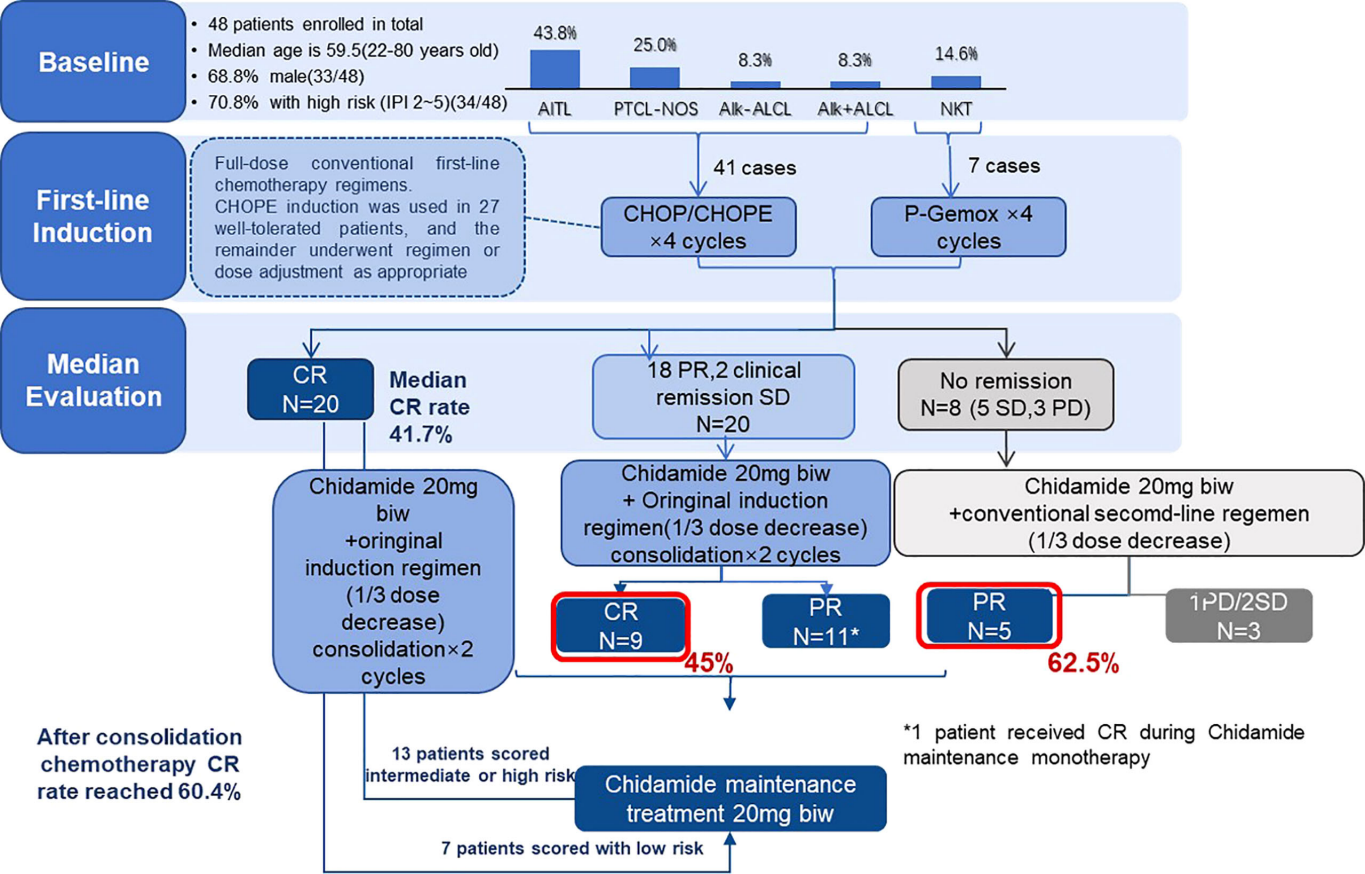
Study design of the whole treatment in 48 patients.

This study was conducted in accordance with the principles of good clinical practice and the Declaration of Helsinki. The protocol, informed consent forms, and other relevant study documentation were approved by the ethics committee of the first hospital of Jilin University (No.2021-681). All patients provided written informed consent before study entry. Detailed study design has been depicted in **Figure 1** and characteristics of participants has been shown in **Table 1**.

**Table 1:** Baseline of the patients (N=48).

Characteristics	Overall (N= 48)		First-line (N=40)		Salvage (N=8)	
	*n*	*%*	*n*	*%*	*n*	*%*
*** Gender***						
**Male : Female**	33: 15	68.8: 31.3	27: 13	67.5:32.5	6: 2	75: 25
*** Age***						
**Median**	59.5 (22~80)		60 (30~80)		50 (22~70)	
***Pathological Subtype***						
**AITL**	21	43.8%	18	45.0%	3	37.5%
**PTCL-NOS**	12	25.0%	8	20.0%	4	50.0%
**NKT**	7	14.6%	6	15.0%	1	12.5%
**ALK+ALCL**	4	8.3%	4	10.0%	0	0.0%
**ALK-ALCL**	4	8.3%	4	10.0%	0	0.0%
*** IPI Score***						
**0~2**	31	64.6%	26	65.0%	5	62.5%
**3~5**	17	35.4%	14	35.0%	3	37.5%

PTCL-NOS, peripheral T-cell lymphoma-not otherwise specified; AITL, angioimmunoblastic T-cell lymphoma; ALCL, anaplastic large cell lymphoma; NKT, NK/T-cell lymphoma.

### 2.2 Treatment and Procedures

Patients received chidamide 20 mg alone twice a week as maintenance after CR1 or in combination with previous regimen as consolidation therapy after PR1 or SD1 (most patients received two combination regimens) (Figure1). CR1 with IPI≥2, chidamide combined with the same regimen but dosage Reduced by 1/3; PR1, chidamide combined with the same regimen in full dosage; SD1, chidamide combined with second line regimen like ICE. The P-GemOx (pegaspargase 2000–2500 IU/m2 [maximum, 3750 IU]) day 1, gemcitabine 1000 mg/m2 days 1 and 8, oxaliplatin 130 mg/m2 day 1). CHOPE/CHOP like (cyclophosphamide 750 mg/m2 day 1, doxorubicin 50 mg/m2 day 1, vindesine sulfate 3 mg/m2 day 1, prednisone 100 mg days 1–5). Three weeks constituted a treatment cycle with the evaluation of efficacy. Starting with chidamide, response assessments were performed by clinical examination and CT/ultrasound every two cycles. All responses were determined using the International Harmonization Project revision of the International Working Group criteria (13). Patients continued treatment until disease progression or unacceptable toxicity. Patients who achieved CR, PR, or SD continued to receive chidamide maintenance treatment.

During the two courses after the initial efficacy evaluation, all patients completed the original chemotherapy regimen combined with oral chidamide. After two consolidation courses, all patients without progressive disease (PD) received maintenance treatment.

### 2.3 End Points and Assessments

The primary endpoint was progression-free survival (PFS) from the time of chidamide initiation. Secondary endpoints were OS and safety. Safety assessments included physical examinations, adverse event (AE) monitoring, and laboratory parameter changes. AEs were graded using the National Cancer Institute Common Toxicity Criteria for Adverse Events scale, version 4.0.

### 2.4 Statistical Analysis

The data cutoff date for this report was August 20, 2021. Descriptive statistics are presented as numbers and percentages for dichotomous/categorical data. The chi-squared test was used for comparisons of categorical variables. The Kaplan–Meier method and log-rank test were employed for survival analysis. Multivariate analysis for OS was performed using the Cox proportional hazards model. All data were analyzed by SPSS25.0.

## 3 Results

### 3.1 Baseline Characteristics

In total, 45 patients were enrolled from March 2016 to May 2021 at the First Hospital, Jilin University (Changchun, China). The baseline characteristics of evaluable patients are presented in **Table 1**. In total, 68.8% of patients were male, and the median patient age was 59.5 years (range, 22–80). The PTCL subtypes were AITL (21/48, 43.8%), PTCL-NOS (12/48, 25%), NK/T-cell lymphoma (NKT, 7/48, 14.6%), and ALCL ALK+/- (4/48, 4/48, 8.3%). 17 patients had intermediate or high risk (IPI = 3–5, 35.4%) at study entry. 7 patients (IPI 0-1) received chidamide as maintenance therapy after achieving CR, whereas 38 patients started chidamide combination regimens as consolidation therapy before transitioning to oral maintenance therapy (13 cases CR1 with IPI≥2; 20 cases PR1; 5 cases from SD1). In total, 57.1% (16/28) of patients achieved better responses during chidamide therapy (PR to CR, nine patients; SD to PR, seven patients).

### 3.2 Treatment

The number of months from the first diagnosis to Chidamide was administered ranged from 0 to 7 months, mainly concentrated in the range of 2.8 to 4.2 months, accounting for 58.3% (28/48). 75% (36/48) patients were administrated initially with Chidamide in C5. All patients with NKT received the P-GemOx regimen as the first-line treatment. Patients with all other subtypes received the CHOPE regimen. The efficacy evaluation was completed before starting the fifth course of treatment.

All 48 patients were initially evaluated as non-drug–resistant (CR/PR/SD). After the initial efficacy evaluation, all patients completed two courses of the original chemotherapy regimen combined with oral chidamide. After two consolidation courses, all patients without progressive disease (PD) received maintenance treatment.

Among the patients, the mean duration of therapy was 12.8 months (range, 3.0–66.6), and 34 (70.8%) patients are still taking chidamide to date. The reasons for treatment discontinuation included disease progression (N = 14).

### 3.3 Remission

All 48 patients underwent an evaluation of remission. The CR rate and ORR were 60.4% and 93.8%, respectively. The best CR rate of 100% was observed for ALK+ ALCL. Regarding other subtypes, 21/21 patients with AITL, 10/12 patients with PTCL-NOS, and 4/4 patients with ALK− ALCL, 6/7 NK/T had a best response of CR/PR (**Table 2**) during the follow-up period. Meanwhile, the CR and ORR did not differ by age (<60 *vs* ≥60: 50.0% *vs* 70.8%, P = 0.091; and 91.7% *vs* 95.8%, P = 0.551) or IPI (<3 *vs* ≥3: 58.1% *vs* 64.7%, P =0.653; and 61.3% *vs* 94.1%, P =0.014) (**Table 3**).

**Table 2:** CR and ORR by pathologic subtype during chidamide therapy [N (%)].

	AITL	PTCL-NOS	NKT	ALCL		Total	P value
				ALK-	ALK+		
**CR**	13 (61.9)	4 (33.3)	6 (85.7)	3 75)	4(100)	30 (62.5)	0.053
**ORR**	21 (100)	10 83.3)	6 (85.7)	4 (100)	4(100)	45 (93.8)	0.681
**Total**	21	12	7	4	4	48 (100)	

Data are presented as N (%).

CR, complete response; ORR, overall response rate; AITL, angioimmunoblastic T cell lymphoma; PTCL-NOS, peripheral T-cell lymphoma-not otherwise specified; ALCL, anaplastic large cell lymphoma; NKT, NK/T-cell lymphoma.

**Table 3:** CR rate and ORR by age or IPI during chidamide therapy.

		CR	P	ORR	P
**Age**	<60 (N=24)	12 (50.0)	0.091	22 (91.7)	0.551
	≥60 (N=24)	17 (70.8)		23 (95.8)	
**IPI**	<3 (N=31)	18 (58.1)	0.653	19 (61.3)	0.014
	≥3 (N=17)	11 (64.7)		16 (94.1)	

Data are presented as N (%).

CR, complete response; ORR, overall response rate.

### 3.4 Survival

#### 3.4.1 Estimated Survival

With a median follow-up time of 12.8 months (range, 3.0–66.6) through August 2021, 14 patients developed PD (29.2%), and 10 patients died of uncontrolled lymphoma (20.8%).

#### 3.4.2 Survival by Response

**Figure 2:**
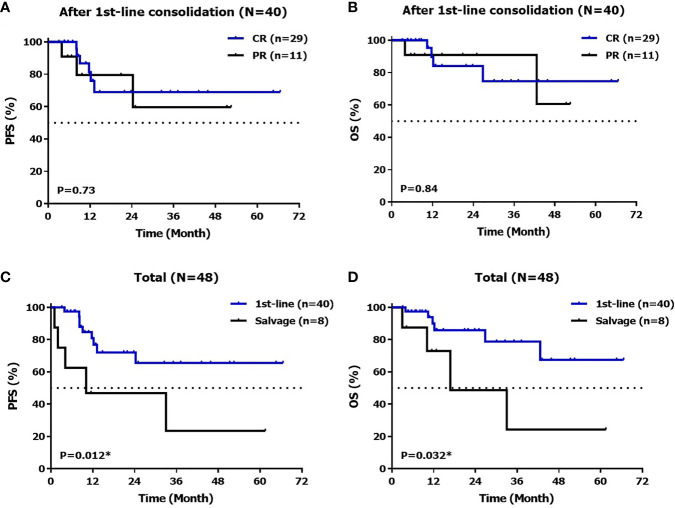
PFS **(A)** and OS **(B)** after 1^st^line therapy, PFS **(C)** and OS **(D)** in total.

PFS did not differ significantly between patients with CR and PR (the 1-year PFS was 81.3% 79.5% and the 2-year PFS was 69% 59.6%, P = 0.73). Whereas differences were noted between patients with first line maintenance (40 cases) and salvage therapy (8 cases) with 1-year PFS 80.8%, 46.9% and 2-year PFS 71.9%, 46.9% (P=0.012), mPFS was not reached *vs* 10.1m, respectively. OS did not differ between patients with CR and PR (the 1-year OS was 89.6%/59.6% and the 2-year OS was 84%/90.9%, P=0.84), but differences were observed between patients with first line maintenance and salvage therapy, 72.9% and 48.6% (P=0.032), mOS was not reached *vs* 16.8m, respectively (**Figure 2**).

Meanwhile, 28 patients did not exhibit CR before starting chidamide treatment, and the curative effect was deepened in 57.1% (16/28) of these patients after treatment, including changes from PR to CR in nine patients (56.3%, 9/16) and from SD to PR in seven patients (43.8%, 7/16), and median PFS and OS were not reached. Moreover, no PD or loss of response was observed during C5 and C6 after starting chidamide. Median PFS was not reached, the 1-year PFS rate, 2-year PFS, 1-year OS rate and 2-year OS rate were 81.3%, 69.0%, 89.6%, 84.0% in patients with complete response, and 79.5%, 59.6%, 90.9%, 90.9% in patients with partial response to chidamide, respectively which have been illustrated in **Figure 2**.

### 3.5 Safety

Chidamide maintenance treatment was generally well tolerated. No patients discontinued treatment because of AEs. The most common toxicities were neutropenia (all grades, 75.0%), anemia (all grades, 79.2%), thrombocytopenia (all grades, 58.3%), anorexia (all grades, 45.8%), and fatigue (all grades, 43.8%) (**Table 4**). The main severe AEs (grade ≥3) were hematologic AEs, especially neutropenia, but only one patient experienced infection (pneumonia). We found that adverse reactions mainly occurred in the first 6 months of chidamide administration, and their incidence decreased during the maintenance period.

**Table 4:** Adverse events (AEs) of chidamide (N=48).

Toxicity Type		*Any grade N (%)*	*Grade ≥ 3 N (%)*
hematologic AEs	Neutropenia	36 (75.0)	16 (33.3)
	Anemia	38 (79.2)	10 (20.8)
	Thrombocytopenia	29 (60.4)	12 (25.0)
	Elevated liver enzymes	15 (31.3)	0 (0)
	Renal dysfunction	6 (12.5)	0 (0)
	Electrolyte disturbance	23 (47.9)	3 (6.3)
non-hematologic AEs	Nausea/vomiting	19 (39.6)	0 (0)
	Anorexia	23 (47.9)	0 (0)
	Fatigue	21 (43.8)	0 (0)
	Pneumonia	1 (2.1)	1 (2.1)

## 4 Discussion

Disease relapse after induction therapy led to poor survival, with a 5-year OS rate of 30%, while patients with relapsed or refractory PTCL had even worse median OS of 5.8 months. These historical data suggested an urgent unmet medical need for PTCL maintenance therapy. Maintenance therapy for PTCL has not been well investigated. To date, only a case report of denileukin diftitox and studies of prolonged treatment with romidepsin and pralatrexate have been reported (7, 14, 15).

In this study, chidamide was used as maintenance therapy either after induction therapy or in combination with consolidation therapy. During a follow-up period of 12.8 months (range, 3.0–66.6), median PFS and OS were not reached, and the 2-year PFS and OS rates were 67.5% and 79.1%, respectively, indicating sustained remission in PTCL. This effect was not inferior to the median PFS of 57.6 months for ASCT after first-line chemotherapy reported in the previous literature (16, 17). The data of this study also illustrated that the treatments were well tolerated. The main adverse reactions occurred within 6 months after initiating chidamide therapy. With the prolongation of maintenance treatment, the incidence of adverse reactions decreased. These data suggested that continuous maintenance treatment with chidamide can improve the efficacy of induction chemotherapy in non-drug–resistant patients, and the efficacy was not inferior to that of first-line ASCT. The efficacy and safety profiles of chidamide as a salvage regimen in relapsed and refractory PTCL were analyzed in a pivotal phase II trial and in real-world experience (10, 18). This study demonstrated for the first time that long-term remission could be achieved *via* chidamide maintenance therapy.

Patients who experienced CR/PR on previous therapies displayed better PFS than those with SD. It is worth noting that although a high proportion of patients (N =28, 58.3%) only achieved PR or SD from previous induction therapies, 16 patients (57.1%) benefitted from continuous treatment and experienced a better curative effect. Median PFS and OS were not achieved at the end of follow-up among such patients in this study, which was significantly better than the response in patients who did not experience a better curative effect. This indicates that continuing chidamide treatment might give rise to PR-to-CR and SD-to-PR/CR conversion and finally long-term remission. Conversely, after the first achievement of CR/PR in this study, continuous chemotherapy combined with chidamide was consolidated. After the curative effect was stable, the treatment strategy of continuous chidamide monotherapy was maintained, which greatly avoided the possibility of recurrence and progression before the introduction of chidamide because of an insufficient depth of remission.

As an epigenetic modulator, chidamide has the ability to potentially regulate the immune system. Studies illustrated that chidamide activates natural killer- and antigen-specific CTL-mediated cellular anti-tumor immunity, and represses Treg expansion. This may result in its activity as long-term maintenance therapy (11, 12).

Of course, this study had some limitations, such as the small number of cases, and some of the patients did not undergo ASCT because of personal choices rather than absolute illness. There are 33 patients younger than 65 years old who did not accept ASCT: 15 patients or their family refuse the high dose chemotherapy; 9 patients could not afford the expenses of ASCT; 4 patients had complications like hepatocirrhosis, Tuberculosis infection, abdominal aneurysm and anxiety disorder; 4 patients were not sensitive to previous chemotherapy; and 1 patients failed on mobilization. These limitations need to be improved in further clinical research.

In conclusion, this study indicated that chidamide maintenance therapy led to potential improvements of PFS and OS with manageable safety profile in patients with PTCL. The strategy of first remission combined with chidamide consolidation chemotherapy and then maintenance treatment is worthy of promotion. Further randomized studies are required to examine the role of chidamide maintenance therapy in PTCL.

## Data Availability Statement

The original contributions presented in the study are included in the article/**Supplementary Material**. Further inquiries can be directed to the corresponding author.

## Ethics Statement

The studies involving human participants were reviewed and approved by The Ethics Committee of the First Hospital of Jilin University. Written informed consent for participation was not required for this study in accordance with the national legislation and the institutional requirements.

## Author Contributions

WG performed data analyses and wrote the manuscript; XW, JL, and XY participated in statistical analysis and graphic production; YZ, YT, and AW collected the raw data; OB conceived and supervised the study. All authors contributed to the article and approved the submitted version.

## Funding

This study was funded by the Natural Science Foundation of Jilin Province, Department of Science and technology of Jilin Province, grant no. 20210101432JC.

## Conflict of Interest

The authors declare that the research was conducted in the absence of any commercial or financial relationships that could be construed as a potential conflict of interest.

## Publisher’s Note

All claims expressed in this article are solely those of the authors and do not necessarily represent those of their affiliated organizations, or those of the publisher, the editors and the reviewers. Any product that may be evaluated in this article, or claim that may be made by its manufacturer, is not guaranteed or endorsed by the publisher.
