# Supplementary figures and images for: Chidamide Maintenance Therapy Following Induction Therapy in Patients With Peripheral T-Cell Lymphoma Who Are Ineligible for Autologous Stem Cell Transplantation: Case Series From China
Source: Front Oncol. 2022 Jun 7;12:875469. doi: 10.3389/fonc.2022.875469 (PMC9209709; doi:10.3389/fonc.2022.875469)

Reasons not suitable for transplantation

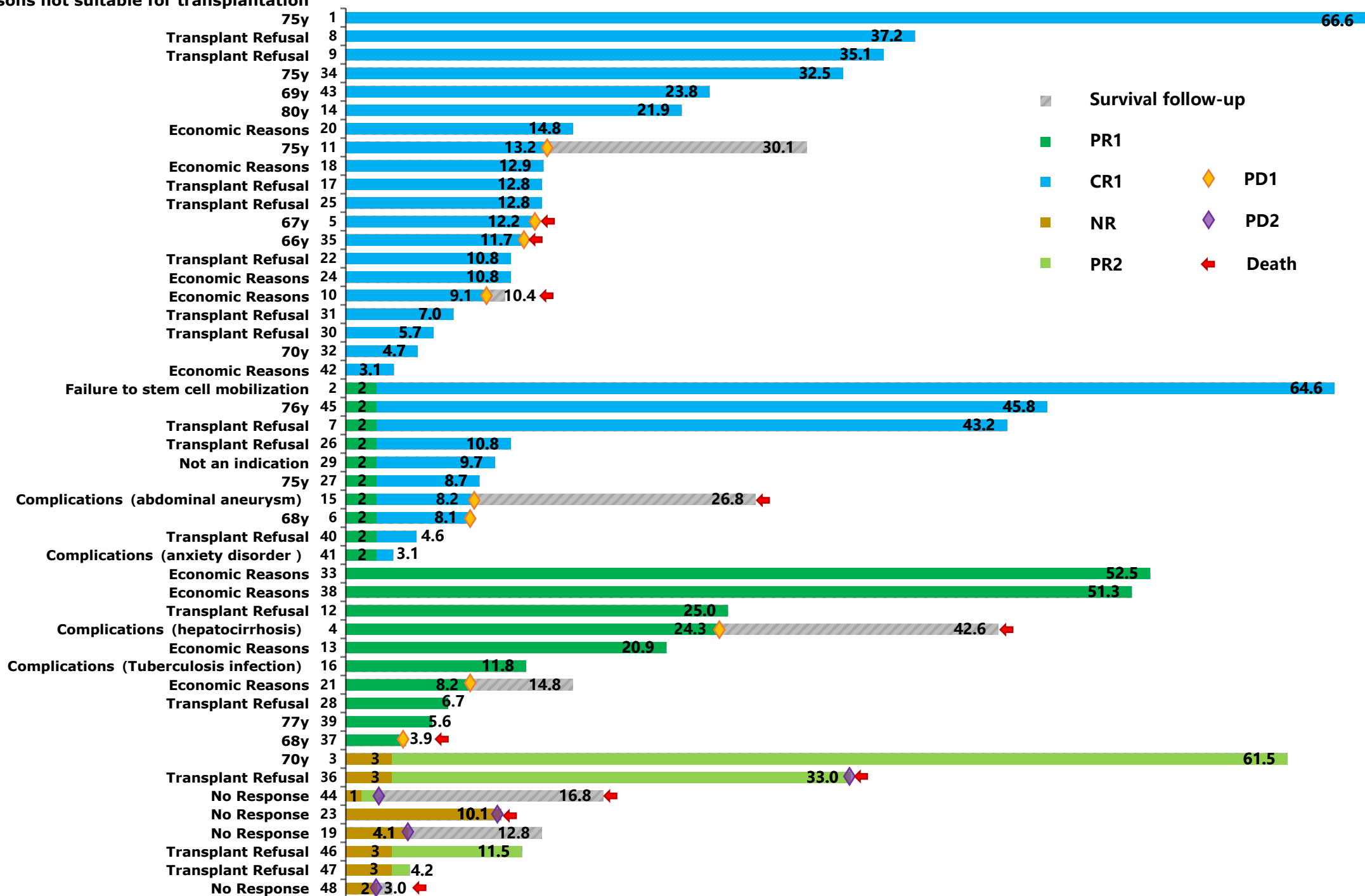

Supplement: Supplementary file 1 [file DataSheet_1.pdf]
